# RNA Sequencing Analysis of the Gametophyte Transcriptome from the Liverwort, *Marchantia polymorpha*

**DOI:** 10.1371/journal.pone.0097497

**Published:** 2014-05-19

**Authors:** Niharika Sharma, Chol-Hee Jung, Prem L. Bhalla, Mohan B. Singh

**Affiliations:** 1 Plant Molecular Biology and Biotechnology Laboratory, Melbourne School of Land and Environment, The University of Melbourne, Parkville, Victoria, Australia; 2 Victorian Life Sciences Computation Initiative, The University of Melbourne, Carlton, Victoria, Australia; Wuhan University, China

## Abstract

The liverwort *Marchantia polymorpha* is a member of the most basal lineage of land plants (embryophytes) and likely retains many ancestral morphological, physiological and molecular characteristics. Despite its phylogenetic importance and the availability of previous EST studies, *M. polymorpha’s* lack of economic importance limits accessible genomic resources for this species. We employed Illumina RNA-Seq technology to sequence the gametophyte transcriptome of *M. polymorpha.* cDNA libraries from 6 different male and female developmental tissues were sequenced to delineate a global view of the *M. polymorpha* transcriptome. Approximately 80 million short reads were obtained and assembled into a non-redundant set of 46,533 transcripts (> = 200 bp) from 46,070 loci. The average length and the N50 length of the transcripts were 757 bp and 471 bp, respectively. Sequence comparison of assembled transcripts with non-redundant proteins from embryophytes resulted in the annotation of 43% of the transcripts. The transcripts were also compared with *M. polymorpha* expressed sequence tags (ESTs), and approximately 69.5% of the transcripts appeared to be novel. Twenty-one percent of the transcripts were assigned GO terms to improve annotation. In addition, 6,112 simple sequence repeats (SSRs) were identified as potential molecular markers, which may be useful in studies of genetic diversity. A comparative genomics approach revealed that a substantial proportion of the genes (35.5%) expressed in *M. polymorpha* were conserved across phylogenetically related species, such as *Selaginella* and *Physcomitrella*, and identified 580 genes that are potentially unique to liverworts. Our study presents an extensive amount of novel sequence information for *M. polymorpha*. This information will serve as a valuable genomics resource for further molecular, developmental and comparative evolutionary studies, as well as for the isolation and characterization of functional genes that are involved in sex differentiation and sexual reproduction in this liverwort.

## Introduction

Bryophytes (e.g., liverworts, mosses, and hornworts) represent an early diverging lineage of embryophytes. Liverworts form the most basal group and evolved as the first land plants to colonize the earth [1]. Thus, liverworts are an important evolutionary clade that may improve our understanding of the genetic mechanisms that allowed plants to evolve from their aquatic algal ancestors and adapt to a terrestrial environment. In addition, liverworts are an ideal group for exploring the following: a) the evolution of dorsiventral development vs. radial development in plants, b) the functions of the genetic network in haploid and diploid generations of land plants and c) the requirement for different developmental programs for body plans in different generations [2]. The exploration of these questions requires the development of genomic resources that facilitate comparative and evolutionary studies.

*Marchantia polymorpha* is a widely distributed dioecious liverwort that is notorious as a weed in damp places and grows well with other plants. The dominant form of this liverwort’s life cycle is a haploid gametophyte thallus that alternates with a short sporophytic diploid phase. *M. polymorpha* reproduces through a sexual mode that involves morphologically distinct male and female sexual organs and by asexual bud-like structures called gemmae, facilitating the active propagation of this liverwort for molecular and biochemical experiments. Despite its evolutionary importance, limited genomic information is available for *M. polymorpha*. With the exception of currently available organellar genomes [3], [4] and the Y chromosome [5] sequences, the nuclear genome of *M. polymorpha* remains unavailable and is currently being sequenced by the Joint Genome Institute (DOE-JGI: http://www.jgi.doe.gov/sequencing/why/99191.html). Because *M. polymorpha* is easily cultivated with available transformation techniques [6], [7], [8], it has become one of the most intensively studied liverwort species. Thus, *M. polymorpha* is an emerging model plant that is appropriate for molecular, comparative and developmental genetics studies.

Previous efforts to perform expressed sequence tags (EST) sequencing, which is a rapid and economical strategy, generated 33,692 ESTs [9], [10], but a detailed description of the genes expressed in *M. polymorpha* remains unavailable. Most of the ESTs were generated from immature male and female sexual organs to identify candidate genes that mediate sex differentiation. Although X and Y chromosome-based markers have been developed [11], functional genomics studies in this liverwort are still in their infancy.

In recent years, the use of next generation sequencing technologies has offered an efficient and cost-effective means of analyzing the transcriptome of an organism via a high-throughput approach [12], [13]. Recent studies have revealed the transcriptomes of a number of model and non-model organisms using next generation sequencing techniques. These studies have been characterized by rapid gene discovery, sensitive gene expression profiling, effective developmental, comparative, functional and evolutionary genomics studies and SNP and SSR marker identification [14], [15], [16], [17], [18], [19], [20], [21], [22]. RNA-Seq generates a large number of sequence reads, allowing global measurement of transcript abundance and providing a deeper and more complete view of the transcriptome without a requirement for prior knowledge of the reference genome [23], [24]. Two methods can be used to construct transcripts from short read sequence data. The first method is the align-then-assemble method, in which short reads are first aligned onto the reference genome sequence, revealing the transcript structure [25], [26]. The second method is the assemble-then-align method, a *de novo* approach, in which transcripts are first constructed through assembly and subsequently aligned to the genome sequence to identify transcript structure [27], [28]. This method is usually used for non-model organisms, which lack genome information, and it requires abundant short read data for accurate assembly.

To our knowledge, this study represents the first comprehensive *de novo* transcriptome assembly from the gametophyte generation of the male and female liverwort *M. polymorpha*. We generated over 80 million sequence reads from six developmental stages of *M. polymorpha* using Illumina RNA-Seq technology. A non-redundant set of 46,533 *de novo* assembled transcripts was generated and subjected to analysis, including GC content analysis, analysis of sequence similarity conservation with other plant species and functional categorization. This work will provide empirical support to the annotated genome sequence when it becomes available, as it contains a dataset of genes with strong transcriptional evidence across a range of tissues and developmental stages. These results also provide a valuable repository of sequence information for the discovery of novel functional genes in *M. polymorpha*.

## Materials and Methods

### Plant Material

Male and female *M. polymorpha* plants were grown in the same conditions as described by Sharma et. al [29]. Tissues were collected from whole gametophytes for RNA sequencing, including three each of male and female developmental stages defined as vegetative thallus, immature reproductive structures (i.e., antheridial and archegonial discs)< = 2 mm in height and mature reproductive structures (i.e., antheridial and archegonial discs) >2 mm in height (Figure S1). The collected tissues were rinsed with distilled water to remove any dirt.

### RNA Extraction, cDNA Library Synthesis and RNA Sequencing

Total RNA was extracted from each of the six above mentioned samples as described by Sharma et. al [29]. On-column DNaseI (Qiagen) digestion was incorporated into the RNA isolation protocol to remove any traces of genomic DNA. RNA isolation steps were performed multiple times, and the RNA collected from each of the six tissues was pooled to meet the starting material requirements for RNA-Seq. This procedure also pools biological replicates in a single run. RNA samples were then quantified and RNA integrity was confirmed using agarose gel electrophoresis. The presence of DNA contamination in the RNA preparations was assessed using agarose gel electrophoresis followed by a PCR reaction that used *MpACT1* gene primers (MpAct1_F: gagcgcggttactctttcacusing MpAct1_R: gaccgtcaggaagctcgtag), Invitrogen *Taq* DNA polymerase enzyme and RNA as the template. High quality RNA samples (40 µg each) were shipped on dry ice to the Beijing Genome Institute (BGI) in China for cDNA library preparation and sequencing. cDNA library construction and paired-end sequencing was performed using an Illumina HiSeq™ 2000 platform (Illumina, San Diego, CA) according to the manufacturer’s instructions as described by Sharma et. al [29].

The generated sequence dataset was deposited at National Center for Biotechnology Information (NCBI) in the Short Read Archive (SRA) database under study accession number SRP029610 (run accession number SRR971243– SRR971249) [29].

### *De novo* Transcriptome Assembly and Measuring Transcript Abundance

Raw reads were quality controlled by removing 3′ adaptor sequences, empty reads and low quality reads (i.e., reads with unknown base ‘N’). Filtering of raw data was performed by BGI in China.

The high quality filtered reads from the 6 tissue samples were then pooled and assembled using the following publicly available programs: Velvet version 1.1.05 (http://www.ebi.ac.uk/~zerbino/velvet/), which was developed for *de novo* short read assembly using de Bruijn graphs [30], and Oases version 0.1.22 (http://www.ebi.ac.uk/~zerbino/oases/), which is a *de novo* transcriptome assembler for very short reads [31]. Computational resources were provided by the Victorian Life Sciences Computation Initiative (VLSCI) in Victoria, Australia (http://www.vlsci.org.au/). After assessing the results obtained with different *k-mer* sizes, a *k-mer* size of 49 was chosen, as it generated the best assembly results with our dataset based on the average contig length and the N50 contig length (see Results and Discussion). Small *k-mers* make the graph very complex, while large *k-mers* have poor overlap in regions with low sequencing depth. Although this higher value reduced the number of assembled contigs, it increased the reliability of longer contigs. After Velvet assembly, the resulting contigs were clustered into small groups, called loci, using Oases to produce transcript isoforms.

Individual library reads were mapped onto the non-redundant set of assembled *M. polymorpha* transcripts using standalone Bowtie version 0.12.7 [32], using same parameters as described by Sharma et. al [29] and were normalized to the RPKM (reads per kilobase of exons per million) [27].

### Assessment of Assembly

The quality and completeness of our *M. polymorpha* transcriptome assembly were assessed in a number of ways, as described below. The assembled set of transcripts was filtered using cd-hit-est from the CD-HIT package [33] with an identity parameter of 95%. This tool was used to cluster similar sequences and remove redundancy in the assembly results.

The completeness of assembly was assessed by determining the number of eukaryotic ultra conserved orthologs (UCOs) [34] present in the *M. polymorpha* transcriptome dataset. UCOs are single copy genes that are common in eukaryotic organisms and are represented in multiple functional categories with a broad spectrum of expression levels. The number of UCOs was determined by comparing the list of 357 UCO coding sequences from *Arabidopsis* (http://compgenomics.ucdavis.edu/compositae_reference.php) to *M. polymorpha* transcripts using NCBI TBLASTX (version 2.2.24) with an E-value cut-off of 1e-05. The BLAST results were parsed to determine the number of UCOs with a positive hit.

The transcriptome assembly was also compared with the predicted proteins from *P. patens,* as downloaded from the PlantGDB database (http://www.plantgdb.org/PpGDB/), using BLASTX with an E-value threshold of 1e-05.

### Functional Annotation of Assembled Transcripts

Local BLASTX with an E-value cut-off of 1e-05 was employed to search known non-redundant protein sequences from NCBI and *Arabidopsis* protein sequences from The Arabidopsis Information Resource (TAIR) (ftp://ftp.arabidopsis.org/home/tair/Proteins/TAIR10_protein_lists/). This search was performed to annotate the assembled *M. polymorpha* transcripts based on sequence comparison with available known protein sequences in public databases. To reduce the search space, we extracted embryophyte protein sequences from NCBI’s nr database and used them for subsequent analysis. The BLASTX results from *Arabidopsis* proteins were used to assign GO terms to transcripts. To assign Kyoto Encyclopedia of Genes and Genomes (KEGG) pathways [35] to our assembled dataset, the KEGGMapper-Search & Color Pathway tool was used with *Physcomitrella patens* as the selected organism and respective gene identifiers for the identification of transcripts associated with KEGG pathways.

The transcripts were also aligned to the Clusters of Orthologous Groups (COG) database (http://www.ncbi.nlm.nih.gov/COG/) and the OrthoMCL database to predict and classify possible functions, accepting the top hit from BLASTX with an E-value threshold of 1e-05 [36], [37]. The program ESTScan was used to detect potential coding regions and to determine the sequence direction in the assembled transcript sequences [38]. Transcript sequences were also searched against NONCODE databank v3.0 [39] to identify non-coding RNA (ncRNA) sequences.

### Comparison with Available EST Sequences from *M. polymorpha* and *Physcomitrella*

A total of 33,692 EST sequences from *M. polymorpha* and 382,587 EST sequences from the closely related species *P. patens* were downloaded from the NCBI database. These sequences were then searched against the assembled set of *M. polymorpha* transcripts using BLASTN with an E-value stringency of 1e-05.

### GC Content Analysis and Detection of Microsatellites

GC content was analyzed using in-house perl scripts for *M. polymorpha*, *Physcomitrella*, *Selaginella*, *Chlamydomonas*, rice and *Arabidopsis* transcripts. The perl script program MISA (MIcroSAtellite) was used to identify SSRs in our dataset (http://pgrc.ipk-gatersleben.de/misa/). The search included the following criteria: repeats of mono-nucleotide were more than 10x, repeats of di-nucleotides were more than 6x, and repeats of tri-, tetra-, penta-, and hexa-nucleotides were more than 5x and present with a minimum distance of 100 bp. The SSR frequency refers to the kilobase pairs of cDNA sequences containing one SSR.

## Results and Discussion

### Sequencing of the *M. polymorpha* Transcriptome

Plant genomics for non-model plants have been a challenge due to large genome sizes, high ploidy levels and the presence of large amounts of repetitive sequences. Transcriptome sequencing may provide a rapid and viable alternative to genome sequencing for characterizing expressed genes [40]. We used high-throughput sequencing data to characterize the gametophyte transcriptome of *M. polymorpha*. Similar to sequencing techniques, a number of bioinformatics tools have also been developed for short-read sequence data assembly and analysis [30], [31]. Although the *de novo* assembly of short reads without a known genomic reference is believed to be troublesome [41], this technique has gathered attention over time [42].

To globally define the gametophyte transcriptome of *M. polymorpha*, RNA samples were collected from six different developmental stages of male and female tissues: vegetative thallus male (VM); vegetative thallus female (VF); immature reproductive male (IMM); immature reproductive female (IMF); mature reproductive male (MM); and mature reproductive female (MF) (Figure S1).

RNA integrity of the RNA samples was confirmed using agarose gel electrophoresis (Figure S2A). To confirm that DNase treatment during RNA extraction was effective and that the cDNA library used for sequencing was free of contaminating genomic DNA, agarose gel electrophoresis was performed using the Invitrogen *Taq* DNA polymerase enzyme, RNA from each of the six tissues as the template and actin (*MpACT1*) gene primers (Figure S2B).

Transcriptome sequencing using the Illumina platform generated 80,936,320 paired-end 90-nt reads, totalling 17 GB of sequence data in fastq format. The raw sequence reads were pre-processed to eliminate primer/adaptor contamination and low-quality sections of reads because these sequences can substantially compromise *de novo* assembly [16]. The data lost during this filtering step is considered to represent an acceptable level of loss to improve assembly accuracy. The final data set used for assembly and analysis of the *M. polymorpha* transcriptome was comprised of high quality reads with a Q20 percentage greater than 93 for each of the six libraries (Table 1).

**Table 1:** Statistics of RNA sequencing.

Tissue	Total Reads	Total Nucleotides (nt)	Q20 Percentage	GC Percentage
Vegetative Thallus Male (VM)	13,878,110	1,249,029,900	93.04%	51.48%
Vegetative Thallus Female (VF)	13,566,404	1,220,976,360	94.16%	50.83%
Immature reproductive Male (IMM)	13,212,124	1,189,091,160	93.62%	50.37%
Immature reproductive Female (IMF)	12,853,456	1,156,811,040	93.76%	50.41%
Mature reproductive Male (MM)	13,554,424	1,219,898,160	93.97%	50.85%
Mature reproductive Female (MF)	13,871,802	1,248,462,180	93.46%	50.90%
Total	80,936,320	7,284,268,800		

Summary of data generated for *M. polymorpha* transcriptome.

All reads in 6 samples were 90 bp in length.

### *De novo* Transcriptome Assembly

**Figure 1:**
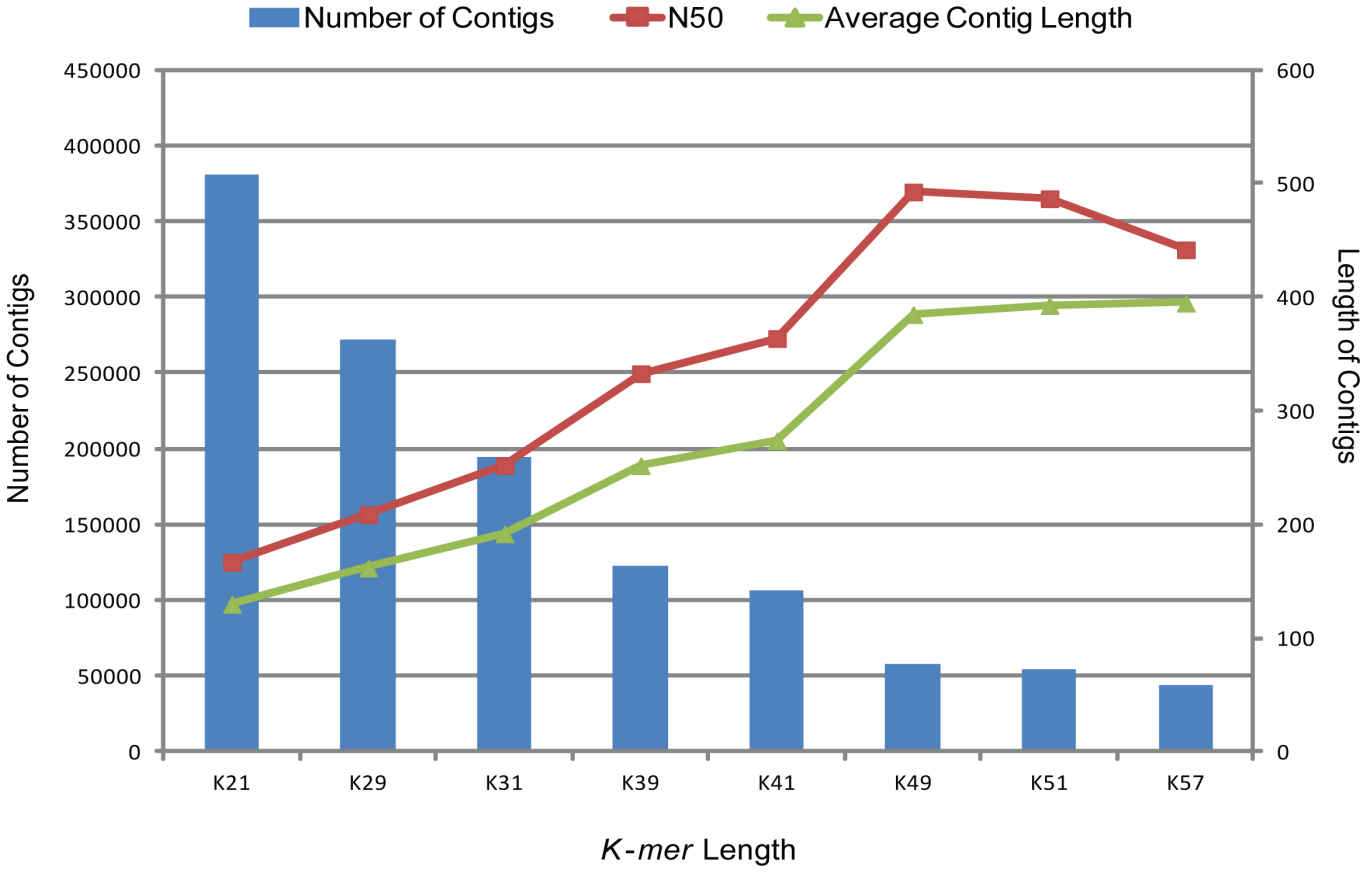
Comparison of *de novo* assembly of VM (vegetative thallus male) data set using Velvet program for different *k-mer* lengths. The bars indicate number of contigs obtained with different *k-mer* lengths used in the assembly program (left axis). The lines indicate N50 length (rectangles) and average contig length (triangles) in bp (right axis).

Due to the absence of a reference genome, *de novo* assembly was performed to construct transcripts from *M. polymorpha* short sequence read data. The assembly of high-quality sequence reads from six tissues of *M*. *polymorpha* was performed using the publicly available programs Velvet (version 1.1.05) [30] and Oases (version 0.1.22) [31]. To determine the optimal *k-mer* length for the data in this study, we first tested a number of different *k-mers* (i.e., 21, 29, 31, 39, 41, 49, 51 and 57) for the Velvet assembly using sequences from the VM library. The assembly results obtained using different *k-mers* were assessed based on the number of reads used, the nodes, the N50 contig length (i.e., 50% of the total assembled sequence was contained in contigs of this length or longer), the total number of contigs, the average contig length and the longest contig length. The result for the VM Velvet assembly with each k-mer (Figure 1) shows that there is a significant effect of *k-mer* length on assembly output. The number of contigs decreased from 381,306 with *k-mer* 21 to 44,282 with *k-mer* 57. The N50 contig length increased from 167 to 493 as *k-mer* length increased from 21 to 49 (Figure 1, Table S1) and decreased with *k-mer* 51. In comparison, the average contig length increased from 130 to 395 bp as the *k-mer* length increased from 21 to 57, although contig length reached saturation at *k-mer* 49. The comparison of assembly results using different *k-mers* suggested an optimal *k-mer* of 49. The longest contig size obtained was 5,595 bp, and 75.3% of reads were used for the assembly.

Finally, the paired-end reads from all six libraries were pooled and complete assembly was performed using Velvet with a *k*-mer length of 49. The sizes of the libraries used for the assembly are presented in Table 1. The assembly process used 74.8% of all reads and resulted in a final dataset of 143,092 contigs, corresponding to 61 MB of sequence data. The N50 contig length, the average contig length and the largest contig were 471 bp, 328 bp and 8,905 bp, respectively (Table 2).

**Table 2:** Statistics of *de novo* assembly using Velvet and Oases.

Reads	
Total number	80,936,320
Total number of paired reads	40,468,160
Number of reads used for assembly	60,590,452
Percentage of used reads	74.86%
**Nodes**	
Total number	256,850
N50	471
Maximum	8,857
**Contigs**	
Total number	143,092
Minimum length	97
Maximum length	8,905
Mean length	328
**Transcripts**	
Total number	46,740
Minimum length	201
Maximum length	9,616
Mean length	757
Number of transcripts > = 200 nt	24,675
Number of transcripts >500 nt	11,830
Number of transcripts >1000 nt	10,235

Details of non-redundant set of *Marchantia* transcripts obtained from Oases assembly.

It has been suggested that assembly with Velvet followed by Oases yields better results [16]. The Oases program was developed specifically for the *de novo* assembly of transcriptomes using short reads. We assembled the contigs generated by Velvet into transcripts using Oases with default parameters. Oases clusters similar contigs into small groups called loci and constructs transcript isoforms using paired-end read information. Thus, we used a two-step assembly strategy to minimize redundancy in our final transcript sequence dataset. Short transcripts (<200 bp) were removed from the final Velvet-Oases results, as the cDNA fragment size for the sequencing library was 200 nt. A total of 46,740 transcripts were obtained, with the largest transcript being 9,616 bp in length (Table 2, Figure S3). In addition, a large proportion of our assembled transcripts (10,235, 21.9%) were 1000 bp or longer. Transcripts of this size are likely to contain full-length CDS. The assembly of this transcriptome could be advantageous for future gene function prediction and functional genomics approaches in liverworts.

To experimentally validate the assembly results, we designed primers and performed RT-PCR and real-time PCR amplifications of a few selected transcripts. In this validation step, nine primer pairs resulted in the amplification of fragments of the expected sizes [29].

### Quantification of *M. polymorpha* Transcripts

**Figure 2:**
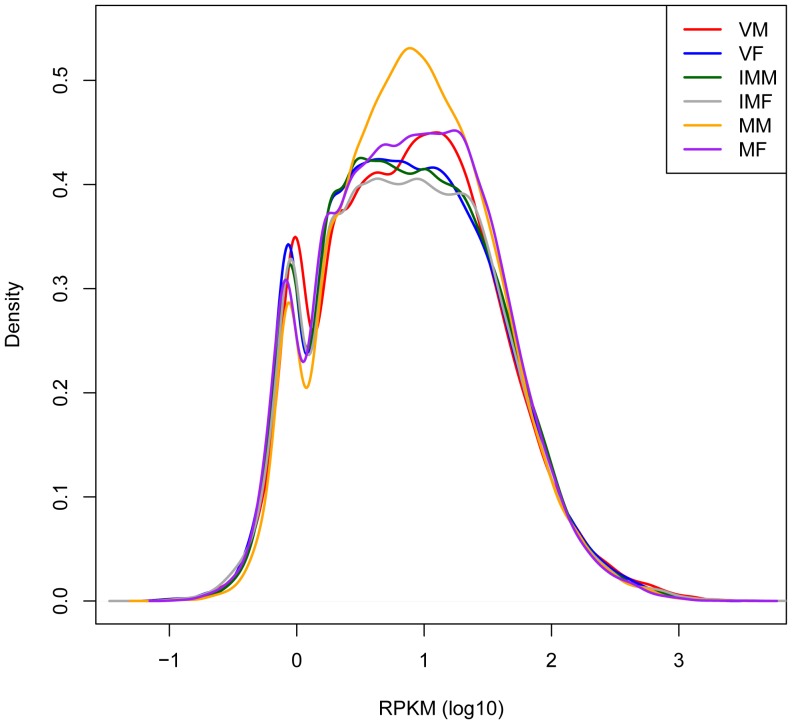
A density plot of RPKM value distribution of *Marchantia* transcripts in six developmental stages. The six stages taken into consideration are VM (vegetative thallus male), VF (vegetative thallus female), IMM (immature reproductive male), IMF (immature reproductive female), MM (mature reproductive male) and MF (mature reproductive female).

The reads from the six different libraries were mapped separately onto the non-redundant set of assembled *M. polymorpha* transcripts using Bowtie version 0.12.7, a short read aligner [32]. On average, 59% of reads were mapped back to transcripts, allowing up to three mismatches per read (Table 3). Gene expression levels can be estimated with high accuracy from RNA-Seq data using the number of reads mapped to the transcripts, and we used RPKM to correct the bias in read count towards longer transcripts [27]. RPKM could be calculated in at least in one sample for only 46,533 transcripts. The remaining 0.5% of transcripts were expressed at undetectable levels for this RNA-seq data or misassembled. These results indicate that the expression of most of the putative genes could be detected in the analyzed tissues and confirm that the employed technique provides sufficient sequence data to survey global gene expression in *M. polymorpha.* The expression levels of the 46,533 transcripts for which RPKM could be obtained were distributed over a wide spectrum, ranging from 0.05 to 13,046, with a mean RPKM of 20.9 (Figure 2, Table S2).

**Table 3:** Mapping statistics of reads from each of the six tissues to the transcriptome assembly.

	VegetativeThallusMale (VM)	VegetativeThallusFemale (VF)	ImmatureReproductiveMale (IMM)	ImmatureReproductiveFemale (IMF)	MatureReproductiveMale (MM)	MatureReproductiveFemale (MF)
**Velvet-Oases result**						
Total Paired Reads	6,939,055	6,783,202	6,606,062	6,426,728	6,777,212	6,935,901
Number of pairedreads mapped	3,601,480	4,062,494	3,937,947	3,898,373	4,077,126	4,311,677
Percentage	51.90%	59.89%	59.61%	60.66%	60.16%	62.16%
Number of pairedreads unmapped	3,337,575	2,720,708	2,668,115	2,528,355	2,700,086	2,624,224
Percentage	48.10%	40.11%	40.39%	39.34%	39.84%	37.84%

Summary statistics of mapping of reads from six cDNA libraries onto the assembled transcripts.

**Figure 3:**
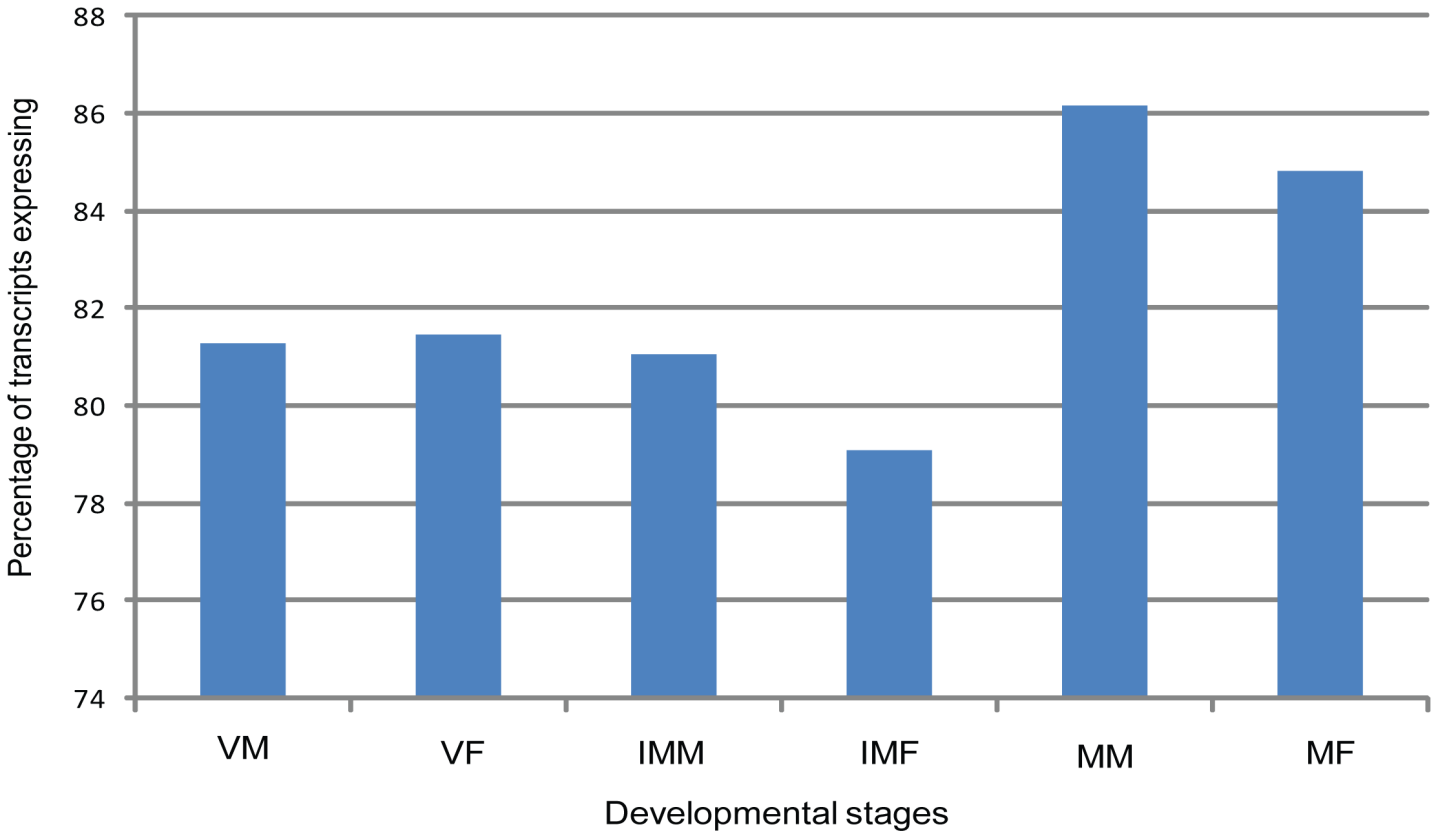
Percentage of transcripts expressed in six developmental stages of *M. polymorpha*. Bar chart showing proportion of transcripts expressing in each of the six libraries to the total transcripts.

We further analyzed the expression patterns of *M. polymorpha* transcripts in six developmental stages: VM, VF, IMM, IMF, MM and MF. The mapping of reads on the *M. polymorpha* transcriptome dataset revealed that 8,706 and 8,638 transcripts are not represented in vegetative tissues (VM and VF, respectively) and that 8,826 and 9,740 transcripts did not align to sequence reads from immature reproductive tissues (i.e., IMM and IMF, respectively). These results indicate that fewer transcripts were expressed in immature reproductive stages than in vegetative stages in *M. polymorpha*. The number of transcripts that were not aligned to mature reproductive tissue sequence reads (i.e., MM and MF) were 6,444 and 7,057, respectively (Figure 3, Table S3). This finding implies that the highest numbers of transcripts are expressed in mature reproductive stages, which signifies the need for a diverse transcript population during the development of reproductive organs.

**Figure 4:**
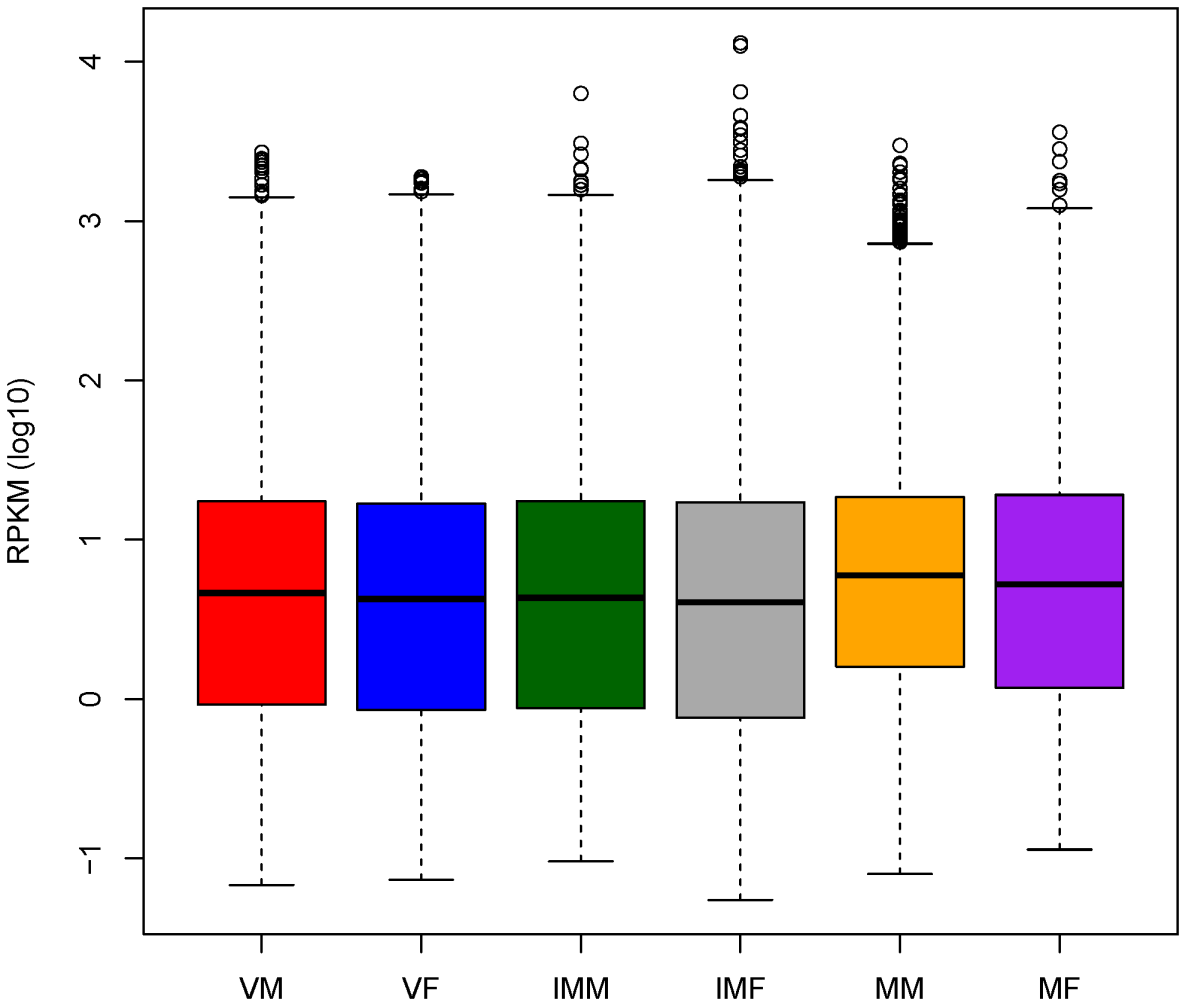
Boxplots of abundance of transcripts in six developmental stages. Boxplots of RPKM values in log_10_-scale in different tissues showing the distribution of these values about the median.

The representation of transcripts from each of the six tissues in the assembled transcriptome of *M. polymorpha* was analyzed in terms of RPKM values. The variation about the median for RPKM values was uniform for all tissues, and the overall contribution of read data from each of the cDNA libraries towards the assembly was similar (Figure 4). The median RPKM values across all tissues were nearly equal, but a proportion of transcripts was very highly expressed in immature stages of reproduction, as indicated by the difference in the 75^th^ percentiles and maximum values for the respective tissue samples (Table S4). Some of these transcripts that are highly expressed in the immature stages of reproduction in liverworts are thought to be involved in sex differentiation processes and the formation of sex organs. A set of 28,381 transcripts was expressed in all stages, and these transcripts may represent the essential genes for this liverwort that play a role in overall development.

Based on the percentage of mappable reads from six different tissues and the distribution of transcript abundance values (i.e., RPKM values), we can conclude that the contribution of reads to the assembly does not appear to be skewed to any one tissue. In summary, we generated the expression profiles of 46,533 transcripts from 46,070 loci using approximately 80 million sequence reads. The 46,533 transcripts represent approximately 37 Mb (13.2%) of the estimated *M. polymorpha* genome [43].

### Assessment of Assembly

The quality and completeness of our *M. polymorpha* transcriptome assembly was assessed in three different ways: by determining the redundancy of assembled transcripts using the CD-HIT package, by comparing the transcriptome to *Physcomitrella* protein sequences and by comparing the transcriptome to a list of UCO coding sequences from *Arabidopsis*.

CD-HIT-EST compared the sequences of assembled transcripts and grouped them into 46,060 clusters. This result is comparable to the 46,070 loci obtained from the Velvet-Oases results, suggesting that the transcript dataset was already nearly non-redundant. We used the Velvet-Oases assembly results for further analysis.

The assembled *M. polymorpha* transcripts were compared to *Physcomitrella* protein sequences using BLASTX with an E-value stringency of 1e-05. The comparison revealed that 18,560 transcripts (39.8%) had hits. The low number of hits could be due to the presence of liverwort-specific transcripts, as liverworts and mosses represent separate clades in the phylogenetic tree [2]. Of the 18,560 transcripts that had hits, 735 (3.9%) had a match with >90% identity, and 5,042 (27.1%) had a match with >70% identity (Table S5). This high degree of sequence similarity with a closely related species indicates that the *de novo* assembly was accurate.

We were also able to identify UCOs, which are *Arabidopsis* genes that are conserved across all eukaryotes and are used as an indirect indicator of the gene sampling breadth, in the assembled *M. polymorpha* dataset. Out of 357 *Arabidopsis* UCOs, 355 (99.4%) were assigned a *M. polymorpha* homologue (Table S6), suggesting that our RNA-seq data detected the expression of the vast majority of *M. polymorpha* genes. Taken altogether, these analyses suggest that the obtained *M. polymorpha* protein-coding transcriptome is a broad representation of the plant’s gene expression potential.

The *Arabidopsis thaliana* genome has recently been shown to have unsuspected transcriptome complexity due to alternative splicing. A total of 57,447 transcripts representing 23,901 genes were detected by RNA-Seq, and more than 60% of the genes that contained introns displayed some form of alternative splicing [44]. Similarly, we obtained 46,533 transcripts with an estimated number of total genes in the *M. polymorpha* genome of approximately 20,000 [43].

### Functional Annotation and Characterization of the *M. polymorpha* Transcripts

**Figure 5:**
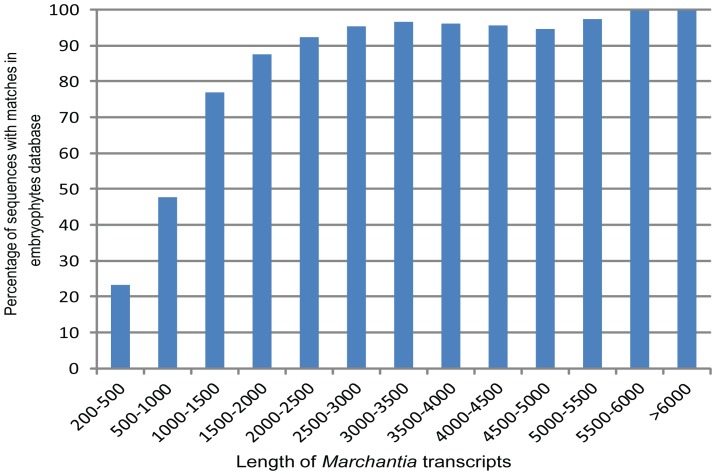
Effect of query sequence length on the percentage of sequences for which significant matches were found. The proportion of sequences with matches (e-value cut off 1e-05) in NCBI embryophytes nr database is greater among longer assembled transcripts.

Functional annotation was performed, and putative functions of transcripts were identified using BLASTX results comparing *M. polymorpha* transcripts to embryophyte proteins extracted from the NCBI nr database and *Arabidopsis* proteins obtained from TAIR with an E-value cut-off of 1e-05. A total of 20,000 (43%) *M. polymorpha* transcripts had significant hits with embryophyte proteins (Table S7). The remaining 26,533 transcripts have a higher proportion of short transcripts (<500 bp), suggesting that BLASTX hits for these short transcripts might not be detected because they do not contain a CDS. Results indicate that the proportion of sequences with matches in the nr embryophyte database is greater for longer assembled sequences (Figure 5). Specifically, among the transcripts longer than 2,000 bp, 90% had BLASTX results in the embryophyte protein database, and this proportion further increased for groups of longer transcripts. In contrast, the proportion of transcripts with BLASTX hits was reduced to below 50% for transcripts shorter than 1,000 bp and further decreased to 23% for those shorter than 500 bp. Because we only compared the transcript sequences with known protein sequences, putative non-coding transcripts may well account for a substantial fraction of the transcripts without BLASTX hits. The identity distribution of top hits in the embryophyte nr database indicated that 14.1% of the sequences have sequence identity higher than 80%, while 85.8% of the hits have sequence identity between 19% and 80% (Figure S4). A total of 170 distinct *M. polymorpha* transcripts had 100% identity with *M. polymorpha* genes that are already available in the NCBI database. Thirty-seven transcripts showed 100% identity with predicted proteins from *Physcomitrella*.

**Figure 6:**
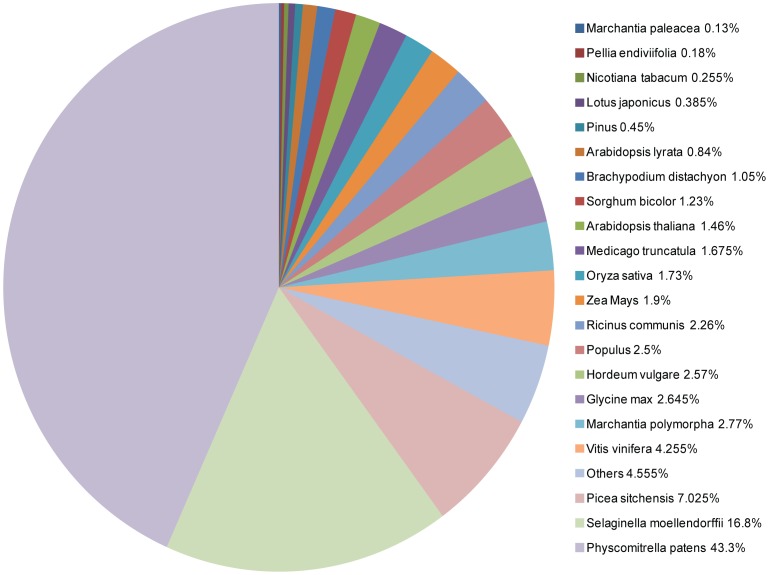
Species distribution of BLASTX top hits to assembled *M. polymorpha* transcripts in embryophytes nr database. Species distribution is shown as the percentage of total homologous sequences with an e-value of at least 1e-05. We used the first top hit of each sequence for the analysis.

In a broad context, the putative orthologs of genes involved in a variety of pathways and cellular processes were conserved in *M. polymorpha*. As expected, the assembled transcripts had a maximum number of top matches with sequences from the moss *Physcomitrella patens* (43.3%), followed by *Selaginella* (16.8%) (Figure 6). The number of hits is not directly related to the relatedness between species, but it is affected by the scope and annotation of available sequence data. As a result, transcripts only showed 2.7% and 0.1% hits with available sequences from *M. polymorpha* and *Marchantia paleacea,* respectively. Many transcripts showed hits with uncharacterized proteins (i.e., 356 uncharacterized proteins, 1,783 unknown proteins, 3,899 hypothetical proteins, 552 unnamed protein products and 8,833 predicted proteins).

The analysis of sequence conservation facilitates the transfer of knowledge from model plants to *M. polymorpha* for functional genomics studies. The lower similarity of *M. polymorpha* transcripts to dicot proteomes than to monocot proteomes is also expected due to the phylogenetic divergence of monocots and dicots during evolution. A significant number of transcripts were found to be conserved only in bryophytes (i.e., shared between *M. polymorpha* and *Physcomitrella*) and may perform bryophyte-specific functions. Interestingly, 57% of the transcripts did not show significant homology with any sequences in the NCBI nr database, which may be due to a lack of annotation in closely related sequenced species. This set of transcripts may also include novel and species-specific transcripts. Lineage and species-specific genes have also been identified in other plant species [45]. The study of these genes will be important for an improved understanding of lineage- and species-specific cellular processes and for the study of evolutionary processes such as speciation and adaptation.

The over-representation of a few functional categories might indicate specific functions or pathways that are operative in liverworts. In addition, 911 *M. polymorpha* transcripts belonged to various functional categories that are conserved in other plants (Supplementary Figure S7).

### Gene Ontology (GO) Assignments and Categorization of Clusters of Orthologous Groups (COGs)

**Figure 7:**
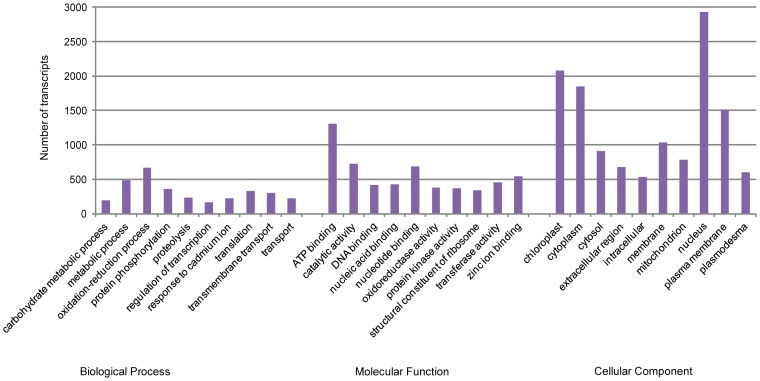
Gene Ontology categorization of *M. polymorpha* transcripts. A bar chart of GOSlim term assigned to the *Marchantia* transcripts in different categories of biological process, molecular function and cellular component.

GO terms were assigned to *M. polymorpha* transcripts that showed significant similarity with *Arabidopsis* proteins (35,386) that were annotated with GO terms. A total of 9,801 transcripts (21%) were assigned to at least one GO term, which was extracted from the top BLASTX hit against *Arabidopsis* proteins using an E-value cut-off of 1e-05. In summary, transcripts were assigned to 18,082 GO terms in the cellular component category, 14,121 GO terms in the molecular function category and 7,639 GO terms in the biological process category (Figure 7, Table S8). Among biological processes, oxidation-reduction processes and metabolic processes were the most highly represented categories. Transcripts involved in other important biological processes, such as transport, response to stress and translation, can also be identified with GO terms. Similarly, transcripts in the molecular function category were heavily dominated by the ATP binding and catalytic activity categories. Most of the transcripts annotated in the cellular component category were localized to the nucleus and chloroplast, but a large number of them were also targeted to the cytoplasm and plasma membrane. Each of the six tissues had a similar GO category distribution for the top 1000 highly expressed loci (Table S9). The dominant GO term categories were the same as those identified for all transcripts.

**Figure 8:**
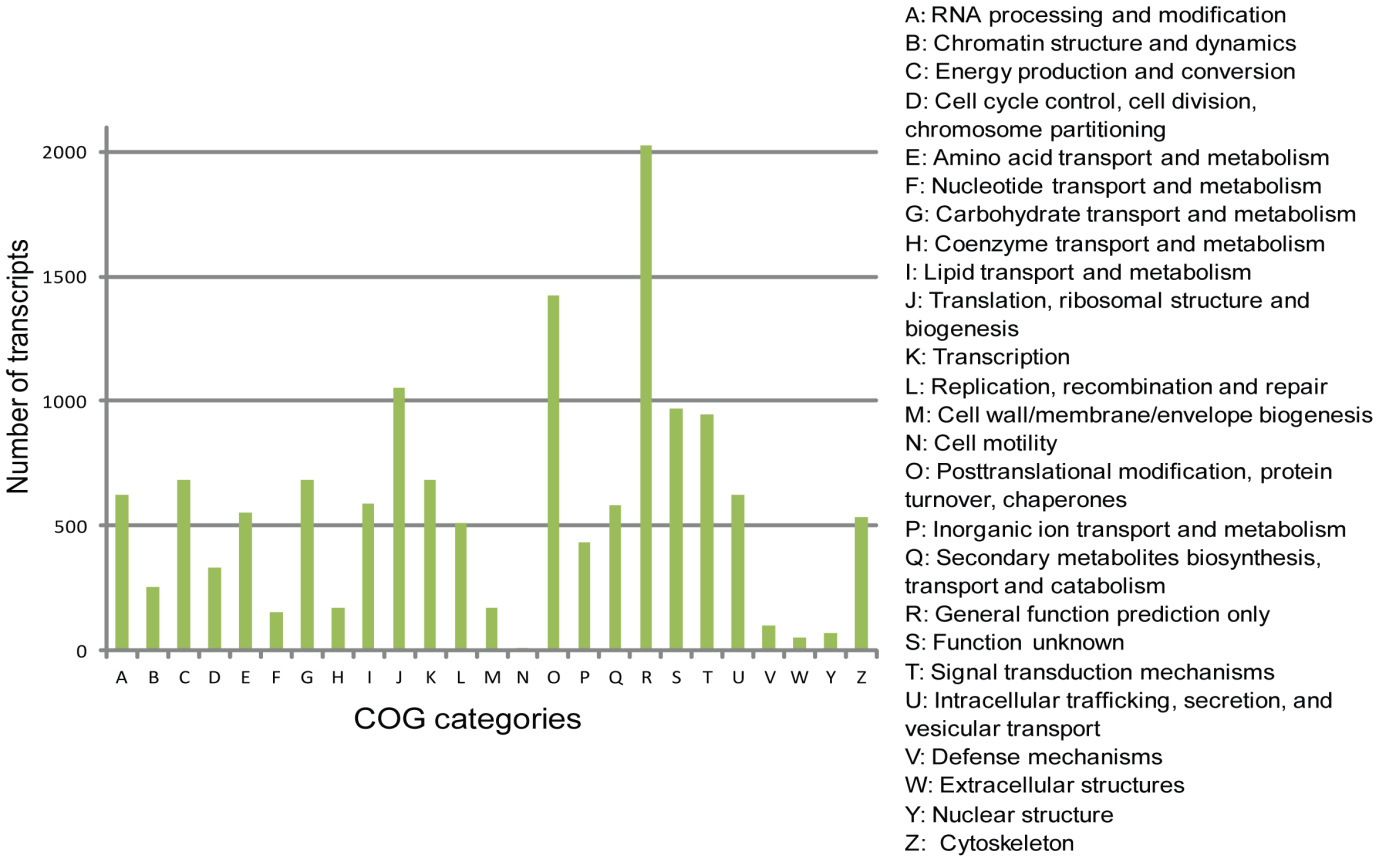
COG classification of *M. polymorpha* transcripts. A histogram of cluster of COG classification of *de novo* assembled *Marchantia* transcripts. 12,687 assembled transcripts were annotated, falling into 25 clusters.

To further elucidate the functional annotation of the assembled transcripts, we analyzed the transcript sequences in the context of different functional classifications in the COG database [36], which classifies orthologous gene products. All proteins present in the COG database are believed to originate from a common ancestor protein, and the dataset contains sequences from species with complete genomes and evolutionary relationships with bacteria, algae and eukaryotes. In total, 12,687 (27.2%) transcripts were categorized into one of the 25 classifications (Figure 8). Of these transcripts, 2,203 belonged to the general function prediction group, which represented the largest group (15.9%) of the 25 categories. The next largest groups were posttranslational modification, protein turnover, and chaperones (1,425 transcripts, 11.2%), translation, ribosomal structure and biogenesis (1,056 transcripts, 8.3%), function unknown (972 transcripts, 7.6%), signal transduction mechanisms (946 transcripts, 7.4%), transcription (684 transcripts, 5.3%), carbohydrate transport and metabolism (683 transcripts, 5.3%), energy production and conversion (681 transcripts, 5.3%), intracellular trafficking, secretion, and vesicular transport (625 transcripts, 4.9%), and RNA processing and modification (624 transcripts, 4.9%). The categories of cell motility, extracellular structures and nuclear structure, which included 13 transcripts (0.1%), 51 transcripts (0.4%) and 71 transcripts (0.5%), respectively, represented the smallest categories. In addition, transcripts belonging to the following categories were also identified: lipid transport and metabolism (589); secondary metabolites biosynthesis, transport and catabolism (580); replication, recombination and repair (510); inorganic ion transport and metabolism (436); cell cycle control, cell division, and chromosome partitioning (331); and defense mechanisms (102).

To further identify the biochemical pathways that are active in *M. polymorpha*, we mapped the transcript sequences that were homologous to *Physcomitrella* sequences to the reference pathways in the Kyoto Encyclopedia of Genes and Genomes (KEGG) [35]. A total of 3,763 transcripts were mapped, and 117 pathways were predicted to be present in *M. polymorpha*. Visual examination of colored KEGG maps demonstrated that the largest represented category is genes required for glycolysis, the citrate cycle and biosynthesis of secondary metabolites, underlying the importance of photosynthesis in plants. We also detected transcripts involved in biosynthesis of amino acids and RNA transport. Of 117 KEGG pathways, metabolic pathways was the largest predicted category. This category contained 720 transcripts involved in carbohydrate metabolism, amino acid metabolism, energy metabolism, nucleotide metabolism, metabolism of cofactors and vitamins and lipid metabolism. Other pathways present in *Marchantia* included the following: spliceosome (88 members); protein processing (87 members); purine and pyrimidine metabolism (79 and 68 members, respectively); oxidative phosphorylation (64 members); RNA degradation (47 members); and endocytosis (34 members). These annotations are valuable resources for the investigation of specific processes, functions and pathways during *M. polymorpha* development.

When all aspects of functional annotation were taken together, the results indicated that these transcripts covered every fundamental biological process, making this transcriptome a comprehensive resource that will benefit subsequent functional genomics studies in *M. polymorpha*. Future studies are required to validate the predicted functions of the transcripts.

### Comparative Genomics

**Figure 9:**
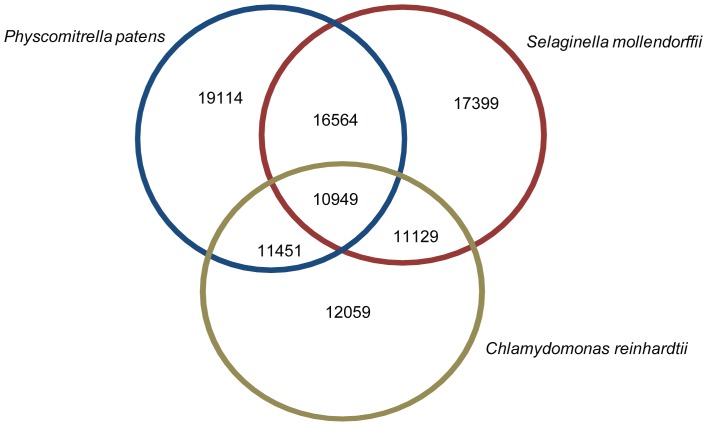
Homologous gene detection in diverse plant transcriptomes. The complete *Marchantia* transcript data set was queried against the complete set of transcripts in the genomes of closely related species *Physcomitrella patens*, *Selaginella mollendorffii* and *Chlamydomonas reinhardtii* using TBLASTX with an e-value cut off of 1e-05. Transcripts with a positive hit in more than one genome are shown in the intersect of those species.

The *M. polymorpha* transcript set was analyzed using TBLASTX to assess similarity and sequence conservation with the transcript datasets for related sequenced species, specifically *Physcomitrella*, *Selaginella*, and the algae *Chlamydomonas*. A total of 10,949 (23.5%) transcripts were shared among all three species. Consistent with the phylogenetic position of these three species with respect to *M. polymorpha*, *Physcomitrella* showed the greatest number of transcript hits (19,114), followed by *Selaginella,* which had hits with 5,340 more transcripts than *Chlamydomonas* (Figure 9). *Physcomitrella* and *M. polymorpha* share 19,114 (41%) transcripts, which can be considered bryophyte-specific genes.

We also analyzed the sequence conservation of *M. polymorpha* transcripts with proteomes from the model dicot *A. thaliana* and the model monocot *Oryza sativa* using a BLASTX search with an E-value cut-off of 1e-05. The results indicated that the percentage of transcripts showing significant homology to monocots is higher than those showing homology to dicots, as 16,590 transcripts (35.6%) showed hits with rice proteins compared to 9,801 transcripts (21%) that showed hits with proteins from dicots.

Using the OrthoMCL tool, 20,072 *M. polymorpha* transcripts were assigned to 8,468 orthogroup MCL clusters (Table S10). The proportion of orthogroups/protein number for each plant is different: 25.2% for *Physcomitrella*, 42.1% for *M. polymorpha*, 41.1% for *O. sativa*, 37.7% for *Arabidopsis* and 57.25 for *Chlamydomonas*.

### Comparison of Assembled Transcripts with EST Sequences from *M. polymorpha* and *P. patens*

The identification of ESTs from the sequencing of cDNA libraries has made a significant contribution to genomics. However, the sequencing of cDNA libraries has time constraints and economic constraints, and the reliability of the results is an issue due to shallow per-base coverage. Next generation deep sequencing techniques overcome these limitations and produce large datasets at a lower cost, allowing for the use of algorithms and bioinformatics tools. Because a cDNA can be sequenced multiple times, self-correcting basecall errors, the accuracy of the results is improved.

To estimate the accuracy of the *de novo* assembly performed in this study, the assembled sequences (46,533 transcripts) were searched against available *M. polymorpha* and *Physcomitrella* EST sequences downloaded from the NCBI database in October 2013. These sequences included 33,692 *M. polymorpha* ESTs and 3,82,587 *Physcomitrella* ESTs. Using an E-value cut-off of 1e-05, only 14,217 (30.5%) transcripts could be aligned to *M. polymorpha* EST sequences and only 577 (1.2%) could be matched to *Physcomitrella* EST sequences. These results indicate that approximately 70% of the *M. polymorpha* sequence data in this study is novel compared to previously available EST data [9], [10].

### Coding Sequence Prediction

The number of predicted partial or complete coding sequences (CDS) was 21,765, representing 46.7% of the total transcripts.

Quality comparisons of assembled transcripts to available protein sequences in public databases and evidence for CDS prediction demonstrated that the transcript assemblies are robust and that thousands of full length CDSs and their respective 5′and 3′ UTR regions were successfully assembled. A comparison of assembled transcripts to gene catalogs of other lower plants and algae by BLAST analysis and functional annotation (i.e., GO and COG categories) indicates that we have sampled an extensive and diverse catalogue of expressed genes that represent a large proportion of the genes expressed in a variety of tissues.

### Prediction of Non-coding RNAs

Non-coding RNA (ncRNA) genes can produce functional RNA molecules without being translated [46], and ncRNA genes can be identified with RNA-Seq data [14]. ncRNAs have been shown to play roles in transcriptional and post-transcriptional regulation and in maintaining genome stability [47], [48]. A BLASTN search of the NONCODE databank [39] indicated that 72 transcripts had significant homology with ncRNAs with putative regulatory functions. NONCODE v3.0 contains 411,554 public sequences from 1,239 organisms covering all kingdoms of life. These 72 transcripts have a low expression level in all six tissues (Table S11). Subsequent studies are required to validate these putative ncRNAs and to ascertain the biological function of these RNA sequences.

### GC Content Analysis of the *M. polymorpha* Transcriptome

**Figure 10:**
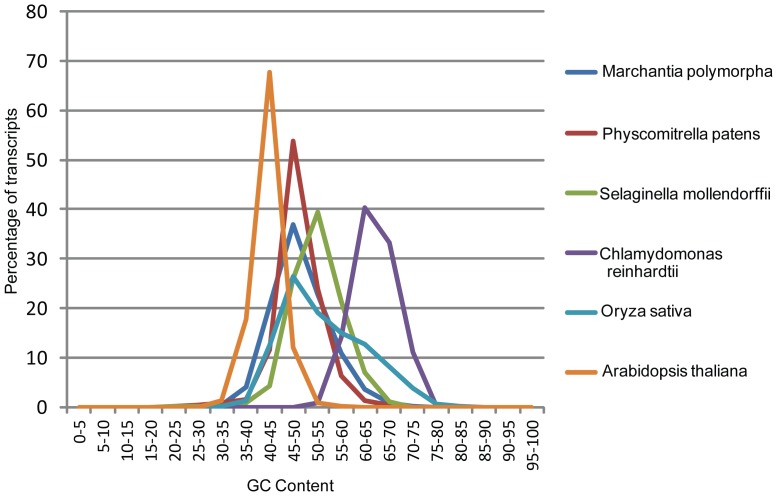
GC content analysis of *M. polymorpha* transcripts. The average GC content of each transcript for *Marchantia*, *Physcomitrella*, *Selaginella*, *Chlamydomonas*, *Arabidopsis* and rice was calculated and percentage of transcripts with GC content within a range are represented.

GC content (i.e., guanine-cytosine ratio) analysis provides insights into a variety of aspects related to the genome of an organism, including evolution, gene structure, thermostability and gene regulation [49], [50]. The GC content of each of the *M. polymorpha* transcripts was calculated and compared with that of *P. patens* (i.e., the closest sequenced reference), *Selaginella moellendorffii* (i.e., the second closest sequenced reference), *Arabidopsis* (i.e., the dicot reference) and rice (i.e., monocot reference). The average GC content of *M. polymorpha* transcripts (48.9%) matched closely with *Physcomitrella* transcripts (48.6%) and was slightly higher than that of *Arabidopsis* (42.1%). The average GC content of transcripts from *Selaginella* (52.5%) and *Chlamydomonas* (64.5%) was much higher and closer to that of rice (53.8%). The average GC content of *M. polymorpha* and *Physcomitrella* transcripts was nearly identical, and these species have the highest proportion of transcripts with GC content in the range of 45–50% (Figure 10). Rice has the highest proportion of transcripts with GC content in the same range. Thus, the overall GC content appears similar for *M. polymorpha*, *Physcomitrella* and rice. The average GC content for the algae *Chlamydomonas* is the highest, and this species was distinct from other species, with GC content in the range of 60–65% (Figure 10). The GC content analysis revealed that *M. polymorpha* transcripts have a low average GC content compared to closely related species and that the GC content of *M. polymorpha* transcripts has a similar distribution to that of monocots.

### Identification of SSRs

Transcript/EST-based markers are an important resource for determining functional genetic variation [51]. Among the various molecular markers, SSRs are highly polymorphic, easier to develop and serve as rich a resource of diversity. To identify SSRs, the *M. polymorpha* transcripts were searched using perl script MISA (MIcroSAtellite) (http://pgrc.ipk-gatersleben.de/misa/). We identified a total of 6,112 SSRs in 4,434 (9.5%) *M. polymorpha* transcripts. Details are presented in Table 4. The tri-nucleotide SSRs represented the largest fraction (52.8%) of identified SSRs, followed by di-nucleotide (28.4%) and tetra-nucleotide (9.1%) SSRs. Mono-nucleotide repeats were less frequent compared to other repeats (6.8%). In addition, only a small number of penta- (160) and hexa-nucleotide (10) SSRs were identified in *M. polymorpha* transcripts. The frequency of identified SSR motifs is also presented (Table S12). The number of tri-nucleotide SSRs was much higher than the number of di-nucleotide SSRs. Previous studies have also reported a higher number of tri-nucleotide SSRs in expressed sequences than genomic sequences. [51] Although the frequency and distribution of SSRs has been proposed to be dependent on many factors, such as dataset size, employed tools and criteria used, the identification of SSRs provides a cost-effective option for developing functional markers.

**Table 4:** Statistics of SSRs identified in *M. polymorpha* transcripts.

SSR Mining	
Total number of sequences examined	46,533
Total size of examined sequences (bp)	35,364,592
Total number of identified SSRs	6,112
Number of SSR containing sequences	4,434
Number of sequences containing more than one SSR	1,133
Number of SSRs present in compound formation	681
**Distribution of SSRs in different repeat types**	
Mono-nucleotide repeat	417
Di-nucleotide repeat	1,737
Tri-nucleotide repeat	2,131
Tetra-nucleotide repeat	557
Penta-nucleotide repeat	160
Hexa-neucleotide repeat	10

Distribution of SSRs found in the assembled transcripts dataset of *Marchantia*.

## Conclusions

In this study we used short read sequence data to rapidly characterize the gametophyte transcriptome from the liverwort *M. polymorpha* for the first time. This study generated a non-redundant set of 46,533 transcripts from this liverwort species, for which genome sequencing is in progress. The detailed dataset analysis revealed several important features of the *M. polymorpha* transcriptome, such as GC content, genes conserved among bryophytes and other related plant species, annotation of functional categories and identification of SSRs. This investigation was aided by the use of *de novo* assemblies that included deep sequencing data from non-model plants, such as chickpea [16], bracken fern [15] and *Nicotiana* [17]. This study delineates the most extensive expressed sequence repertoire available from liverworts to date and provides a substantial addition to the existing EST resource. This data can also be used to develop gene expression assays or used as a reference transcriptome for future RNA-Seq experiments involving sporophytic stages of *M. polymorpha*. The computational prediction of functions for the *M. polymorpha* transcripts in this study provides preliminary information for the predicted genes which may serve as targets to study new designs with old genes [52]. This work significantly expands the genomic resources available for *M. polymorpha* and provides new leads for functional and comparative genomics studies to identify the way of regulatory network recruitment during land plant evolution [53].

## Supporting Information

Figure S1 **Developmental stages of** ***M. polymorpha*** **selected for RNA-Seq.** VM (vegetative thallus male), VF (vegetative thallus female), IMM (immature reproductive male), IMF (immature reproductive female), MM (mature reproductive male) and MF (mature reproductive female). Immature male and female reproductive structures (antheridial and archegonial discs) –2 mm in height and mature male and female reproductive structures (antheridial and archegonial discs) >2 mm in height are taken into consideration for experimental purposes.
(TIF)

Figure S2 A: RNA Gel. RNA integrity was confirmed using agarose gel electrophoresis for RNA samples. First two lanes show RNA samples isolated from Qiagen kit and rest lanes show samples isolated from Trizol. Because Qiagen kit preparation of RNA samples gave better results, Qiagen kit for RNA isolation was used for all RNA isolations from all stages taken into consideration. B: Checking DNA contamination in RNA preparations. Presence of any DNA contamination was checked with agarose gel electrophoresis using Invitrogen *Taq* DNA polymerase enzyme with RNA samples as the template for each of the six tissues with actin (MpACT1) gene primers. MpAct1_F: gagcgcggttactctttcac MpAct1_R: gaccgtcaggaagctcgtag
(TIF)

Figure S3 **Length distribution of assembled** ***M. polymorpha*** **transcripts.** A histogram of transcripts length after 2-step assembly process.
(TIFF)

Figure S4 **Identity distribution of top hits of BLASTX against embryophytes nr database of NCBI.** A pie chart depicting the identity distribution of top hits of BLASTX of *Marchantia* transcripts against the embryophytes nr database.
(TIF)

Table S1 **Assembly statistics of vegetative thallus male (VM) library using Velvet with different** ***k-mer*** **lengths.** A Comparison of *de novo* assembly of VM (vegetative thallus male) data set using Velvet with different *k-mer* lengths.
(XLSX)

Table S2 **RPKM values for all assembled transcripts in 6 tissues.** RPKM values of all assembled transcripts >200 bp in length in six developmental stages taken into consideration.
(XLSX)

Table S3 **Number of transcripts expressed in six developmental stages of** ***M. polymorpha*** **.** Table showing percentage of transcripts expressed and number of transcripts not for which reads are not mapped in each of the six tissues.
(XLSX)

Table S4 **Table used for preparing Box plots depicting concentration of transcripts in six developmental stages.** RPKM values of 46,533 *Marchantia* transcripts are log_10_ transformed and then maximum value, minimum value, 25^th^ percentile and 75^th^ percentile of data is calculated for each of the 6 stages. RPKM 0 was removed before log-transformation.
(XLSX)

Table S5 **Results of BLASTX of** ***M. polymorpha*** **transcripts against predicted proteins in** ***Physcomitrella patens*** **.** Top hits of BLASTX results of 18,560 *Marchantia* transcripts against protein sequences of *Physcomitrella*.
(XLSX)

Table S6 **Results of TBLASTX of** ***M. polymorpha*** **transcripts against UCO genes in** ***Arabidopsis thaliana*** **.** Top hits of TBLASTX results of 355 *Marchantia* transcripts against UCOs in *Arabidopsis*.
(XLSX)

Table S7 **Results of BLASTX of assembled transcripts against nr set of predicted proteins in embryophytes from NCBI.** Top hits of BLASTX results of 20,000 *Marchantia* transcripts against embryophytes nr database of NCBI.
(XLSX)

Table S8 **Hits with** ***Arabidopsis*** **proteins with GO term categorization.** A table showing the *Arabidopsis* homologues of 9,801 *Marchantia* transcripts and their GO terms.
(XLSX)

Table S9 **GO terms obtained for top 1000 highly expressing transcripts in 6 stages.** A table showing the GO terms obtained in 3 categories of cellular component, biological process and molecular function for top 1000 expressing transcripts in each of the 6 stages.
(XLSX)

Table S10 **Assignment of OrthoMCL groups to** ***M. polymorpha*** **transcripts.** A table showing the top hits of BLASTX results of 20,072 *Marchantia* transcripts against OrthoMCL clusters.
(XLSX)

Table S11 **Results of transcripts showing hits with non-coding RNA database.** A table showing the 72 *Marchantia* transcripts that showed significant homology with non-coding RNAs in ncRNA search of the NONCODE databank.
(XLSX)

Table S12 **Statistics of SSRs.** Frequency of identified SSR motifs in *Marchantia* transcriptome dataset.
(XLSX)
