# Supplementary figures and images for: RNA Sequencing Analysis of the Gametophyte Transcriptome from the Liverwort, Marchantia polymorpha
Source: PLoS One. 2014 May 19;9(5):e97497. doi: 10.1371/journal.pone.0097497 (PMC4026138; doi:10.1371/journal.pone.0097497)

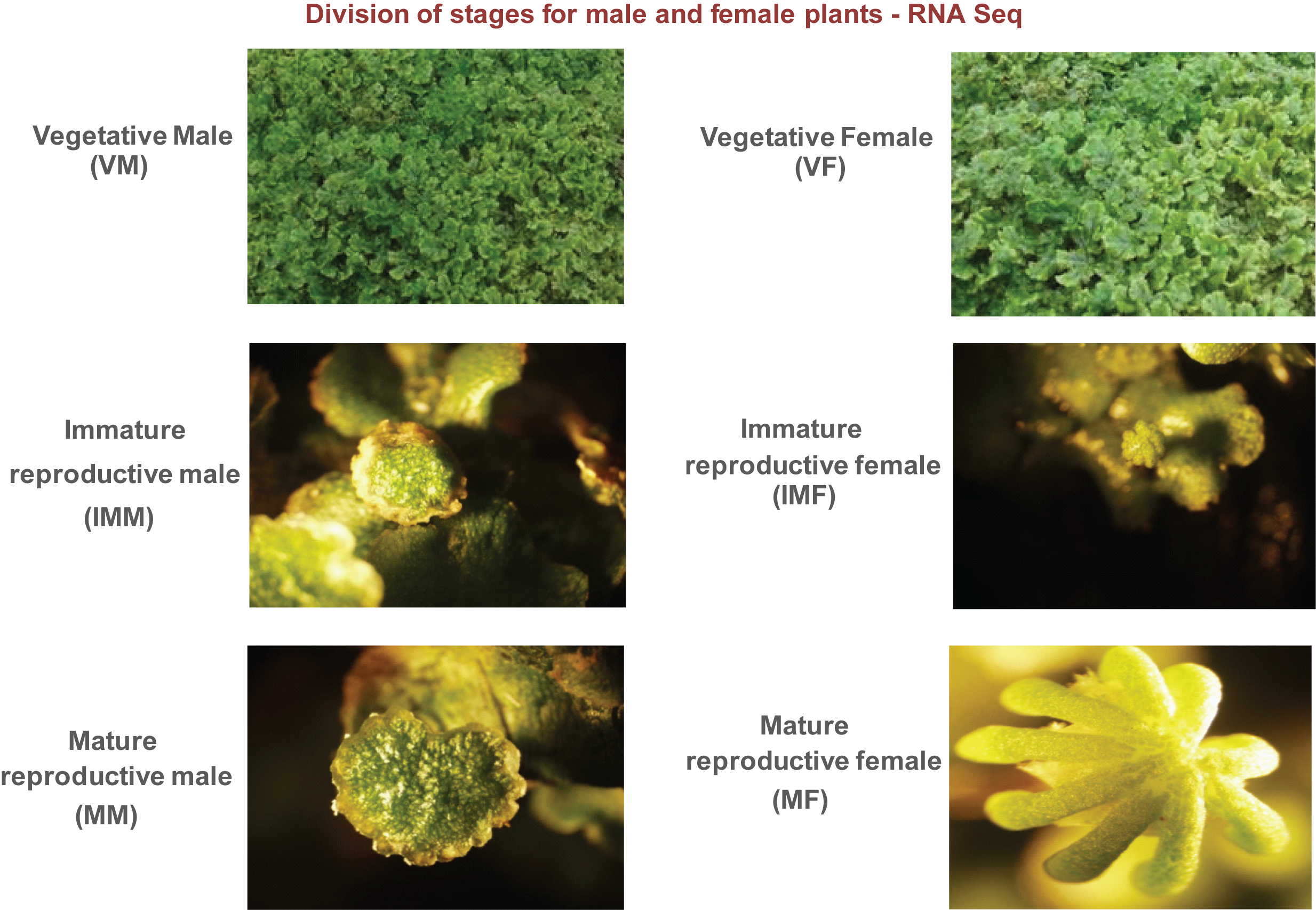

Supplement: Figure S1 — Developmental stages of M. polymorpha selected for RNA-Seq. VM (vegetative thallus male), VF (vegetative thallus female), IMM (immature reproductive male), IMF (immature reproductive female), MM (mature reproductive male) and MF (mature reproductive female). Immature male and female reproductive structures (antheridial and archegonial discs) –2 mm in height and mature male and female reproductive structures (antheridial and archegonial discs) >2 mm in height are taken into consideration for experimental purposes. (TIF) [file pone.0097497.s001.tif]

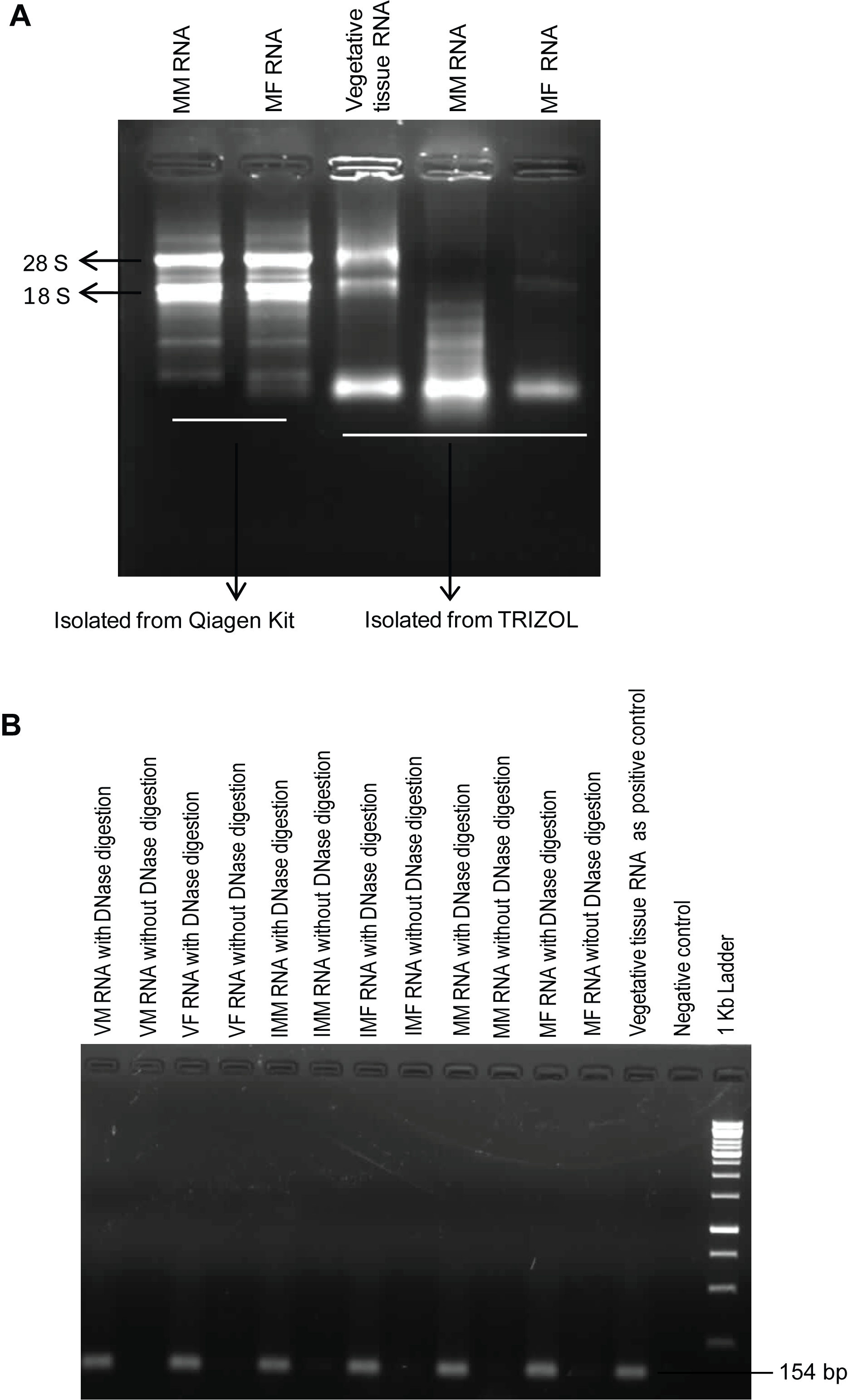

Supplement: Figure S2 — A: RNA Gel. RNA integrity was confirmed using agarose gel electrophoresis for RNA samples. First two lanes show RNA samples isolated from Qiagen kit and rest lanes show samples isolated from Trizol. Because Qiagen kit preparation of RNA samples gave better results, Qiagen kit for RNA isolation was used for all RNA isolations from all stages taken into consideration. B: Checking DNA contamination in RNA preparations. Presence of any DNA contamination was checked with agarose gel electrophoresis using Invitrogen Taq DNA polymerase enzyme with RNA samples as the template for each of the six tissues with actin (MpACT1) gene primers. MpAct1_F: gagcgcggttactctttcac MpAct1_R: gaccgtcaggaagctcgtag (TIF) [file pone.0097497.s002.tif]

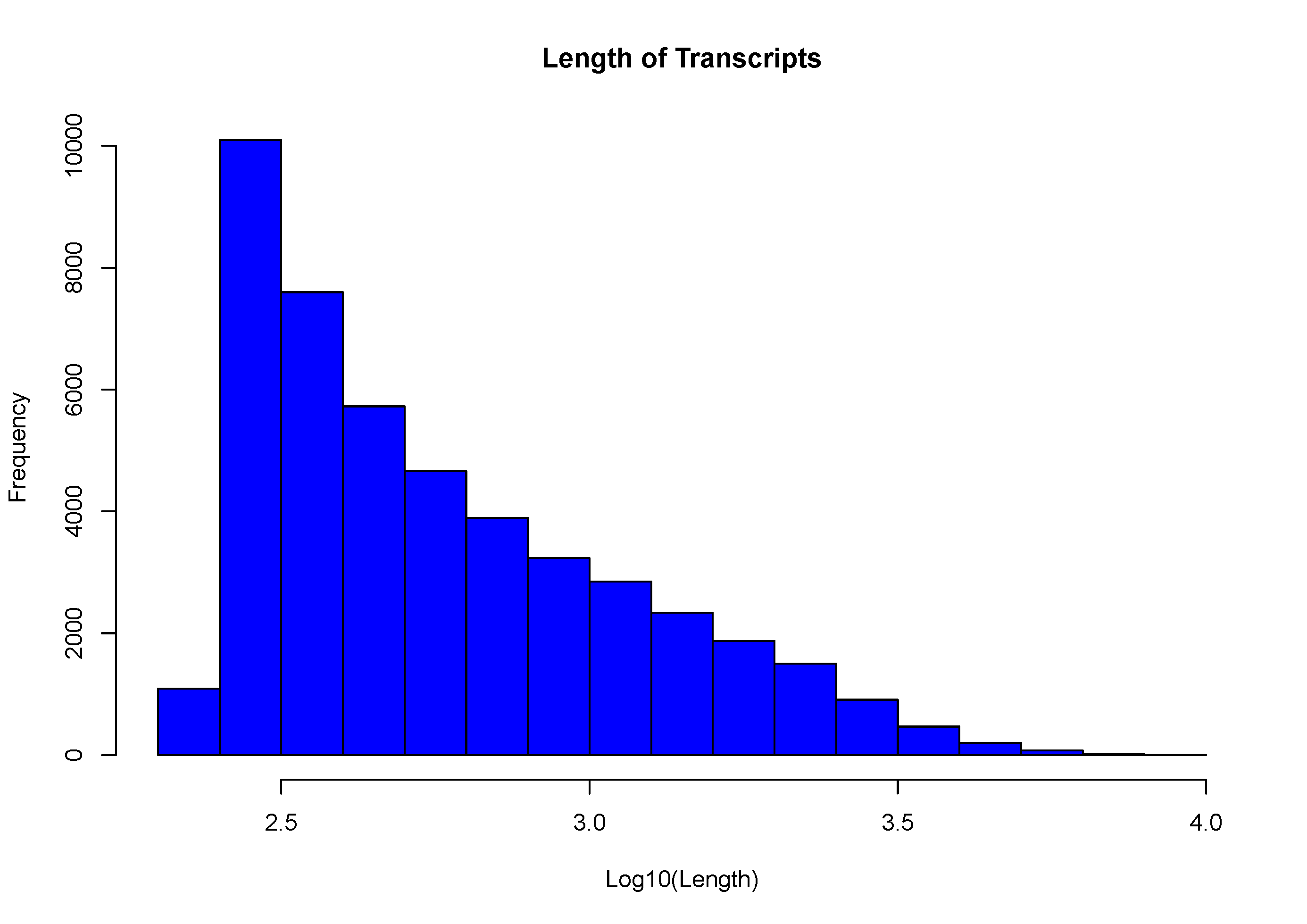

Supplement: Figure S3 — Length distribution of assembled M. polymorpha transcripts. A histogram of transcripts length after 2-step assembly process. (TIFF) [file pone.0097497.s003.tif]

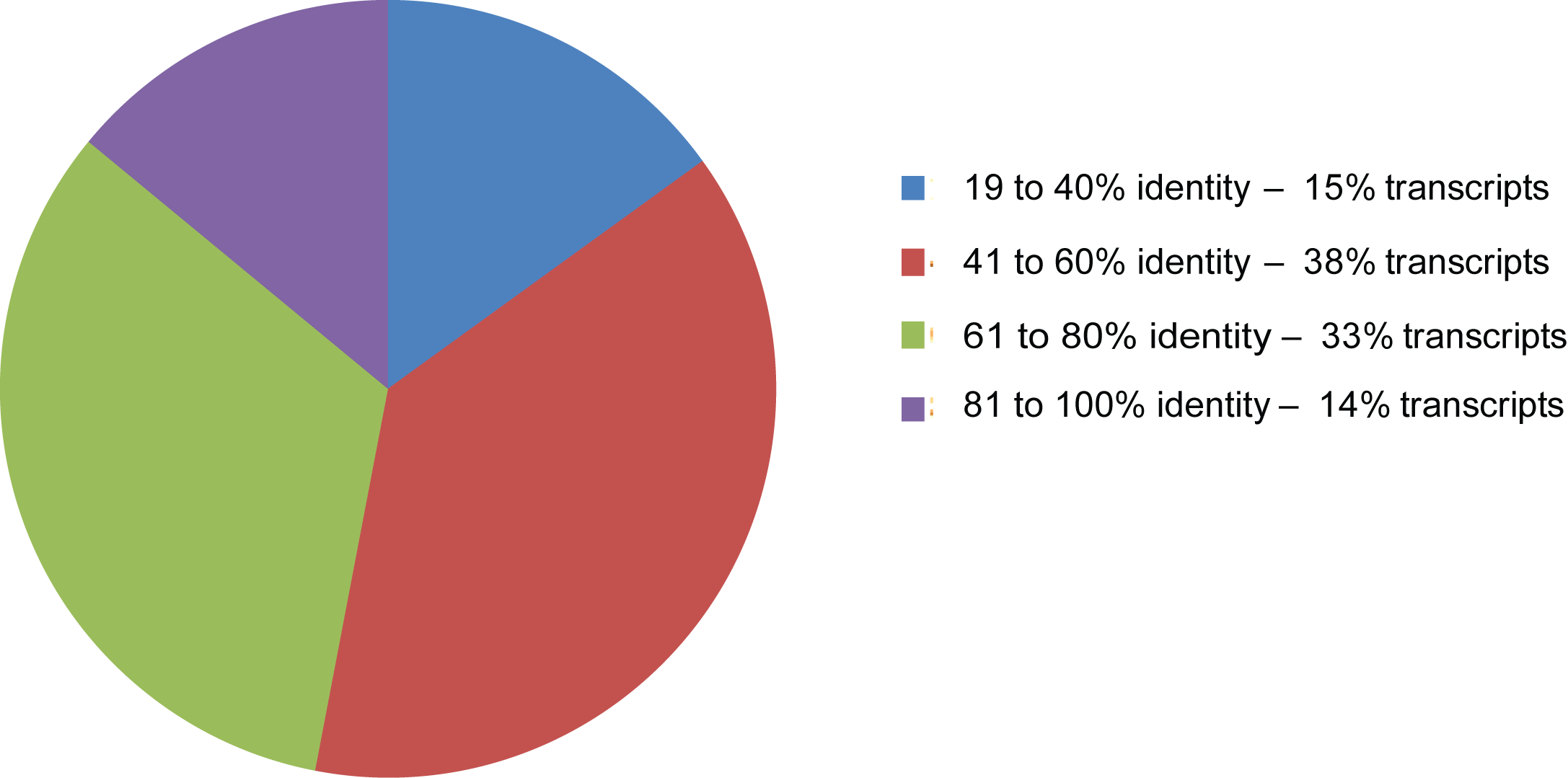

Supplement: Figure S4 — Identity distribution of top hits of BLASTX against embryophytes nr database of NCBI. A pie chart depicting the identity distribution of top hits of BLASTX of Marchantia transcripts against the embryophytes nr database. (TIF) [file pone.0097497.s004.tif]
